# Airborne particle dispersion by high flow nasal oxygen: An experimental and CFD analysis

**DOI:** 10.1371/journal.pone.0262547

**Published:** 2022-01-21

**Authors:** Caroline Crowley, Brian Murphy, Conan McCaul, Ronan Cahill, Kevin Patrick Nolan

**Affiliations:** 1 School of Mechanical and Material Engineering, University College Dublin, Dublin, Ireland; 2 Department of Anaesthesia, The Rotunda Hospital, Dublin, Ireland; 3 Department of Anaesthesia, Mater Misericordiae Hospital, Dublin, Ireland; 4 School of Medicine and Medical Science, University College Dublin, Dublin, Ireland; 5 Centre for Precision Surgery, Section of Surgery and Surgical Specialities, School of Medicine, University College Dublin, Dublin, Ireland; Technion Israel Institute of Technology, ISRAEL

## Abstract

High Flow Nasal Oxygen (HFNO) therapy offers a proven means of delivering respiratory support to critically ill patients suffering from viral illness such as COVID-19. However, the therapy has the potential to modify aerosol generation and dispersion patterns during exhalation and thereby put healthcare workers at increased risk of disease transmission. Fundamentally, a gap exists in the literature with regards to the effect of the therapy on the fluid dynamics of the exhalation jet which is essential in understanding the dispersion of aerosols and hence quantifying the disease transmission risk posed by the therapy. In this paper, a multi-faceted approach was taken to studying the aerosol-laden exhalation jet. Schlieren imaging was used to visualise the flow field for a range of expiratory activities for three healthy human volunteers receiving HFNO therapy at flow rates of 0—60 L/min. A RANS turbulence model was implemented using the CFD software OpenFOAM and used to perform a parametric study on the influence of exhalation velocity and duration on the dispersion patterns of non-evaporating droplets in a room environment. A dramatic increase in the turbulence of the exhalation jet was observed when HFNO was applied. Quantitative analysis indicated that the mean exhalation velocity was increased by 2.2—3.9 and 2.3—3 times that for unassisted breathing and coughing, respectively. A 1—2 second increase was found in the exhalation duration. The CFD model showed that small droplets (10—40 *μm*) were most greatly affected, where a 1 m/s increase in velocity and 1 s increase in duration caused an 80% increase in axial travel distance.

## Introduction

The COVID-19 pandemic has caused major disruption in all sectors of society, but none more so than in the healthcare community. The rapid spread of the coronavirus disease has highlighted the urgent need for a means of delivering respiratory support for critically-ill patients in a safe and effective manner on a large scale. High-flow nasal oxygen (HFNO) therapy has been identified as a potential option [1], offering a less-severe alternative to tracheal intubation in resource-constrained settings [2] and showing increased patient comfort and compliance over conventional oxygen therapy [3]. HFNO has been identified as an aerosol-generating procedure (AGP), meaning its application may change the volume, size, distribution and speed of respiratory particles expelled by patients [4]. Since transmission of the virus occurs primarily through the emittance of these particles from an infected person [5], the potential for HFNO to put healthcare workers at an increased risk of viral exposure has been a major topic of debate in the medical and scientific community [6].

As it stands the evidence to support this hypothesis is limited. Much of the literature comes in the form of retrospective studies on patients in hospital settings [7–9]. The few experimental studies investigating HFNO indicate exhaled air dispersion increases with increasing flow rate applied [10]. However, the environmental contamination associated with the therapy is not significantly increased when compared to conventional oxygen therapy [11]. Modelling techniques have also been employed to support this finding [12].

There is a clear lack of controlled scientific or clinical studies concerning HFNO that urgently needs to be addressed if this treatment is going to be accepted and utilised on a widescale clinical bases. In this work, schlieren imaging is employed to investigate the effect of HFNO on the fluid flow dynamics of the exhalation jet. This technique, using carefully aligned mirrors and light sources, allows the exhaled air to be visualised and has been widely used to study human exhalation with a high degree of success [13, 14]. Additionally, imaging analysis methods allow the obtained images to be quantitatively analysed to extract flow parameters.

Computational Fluid Dynamics (CFD) is a powerful modelling tool that allows analysis of complex fluid flow regimes using a numerical solution based on the governing mathematical equations. The advantages are numerous: Multiple flow parameters can be analysed simultaneously; models can be easily adjusted to simulate different scenarios; and the size of the domain under investigation can be greatly increased in comparison to experimental methods for investigating fluid flow. Since schlieren imaging can only observe the gaseous phases and respiratory droplets are largely invisible to the naked eye, this technique has seen widespread utilisation during the ongoing pandemic to replicate exhalation events and the associated aerosol cloud that can carry the virus [12, 15–20]. CFD is utilised in this work to estimate the effect of HFNO application on the spread of exhaled aerosols.

## Materials and methods

### Experimental set-up

The experimental study was completed in the retired surgical theatres at the Mater Misericordiae University Hospital using schlieren imaging. The temperature difference, and hence density difference, between the exhaled air and ambient room air created a refractive index gradient that allowed the flow field to be visualised. The schlieren optical system was based on two parabolic mirrors of 400 mm diameter and 1.8 m focal length, using a standard razor blade to filter the light beams emitted by a 470 nm LED (ThorLabs) with a diffuser and pin hole. Participants were seated on a surgical bed approximately halfway between the two mirrors, at 90 degrees to the optical axis. The images were captured using a Canon EOS 5D Mark III camera with a Canon EF 100mm f/2.8 Macro lens at 720p60 All-I (with an actual framerate of 59.94 fps). Three participants took part in the study, each receiving HFNO therapy at flow rates of 0, 10, 20, 30, 40, 50, 60 L/min. Participants were given instructions to complete three cycles of breathing, inhaling through the nose and exhaling through the mouth, followed by three distinct coughs. Each exhalation activity was recorded as a separate video file.

An ethical approval ethics exemption was obtained via the UCD Human Research Ethics Committee—[Sciences (HREC-LS)] LS-E-21–129-Nolan received via email notification. Participant consent was informed and agreed verbally and was witnessed by staff at the Mater Misericordiae University Hospital.

### Experimental data analysis

The acquired video clips were processed using a custom-written MATLAB algorithm (MATLAB vR2020b). The purpose of the code was to estimate the direction and magnitude of the exhaled air flow field as revealed by the schlieren technique. The video was firstly pre-processed to remove time-averaged effects to sensitise the data for optical flow. The code implemented then employs the *opticalFlowFarneback* function to create an optical flow object based on the Färneback method [21], in conjunction with the *estimateFlow* function to estimate the optical flow between two consecutive image frames. The algorithm is fundamentally based on comparing positions of features in the video between sequential frames to calculate a velocity vector for each pixel in the image. The mean velocity magnitude at the mouth exit was estimated for each timepoint by averaging the velocity vectors associated with the pixelated region close to the mouth using a moving average filter. This technique was used to analyse all obtained video files.

### CFD model description

The purpose of the CFD model was to allow extrapolation of the exhalation jet based on the results of the experimental study and implement respiratory droplets to the flow. The model represents a small room with an individual standing at one end. The individual releases an exhalation jet laden with respiratory droplets that are subsequently dispersed in the ambient environment. A Eulerian-Lagrangian modelling approach was taken which uses two-way coupling between the bulk carrier phase (ambient and exhaled air) and the discrete phase (respiratory droplets) such that the interaction between the phases is accounted for. The model was implemented using the open source CFD code OpenFOAM.

#### Computational domain

**Fig 1:**
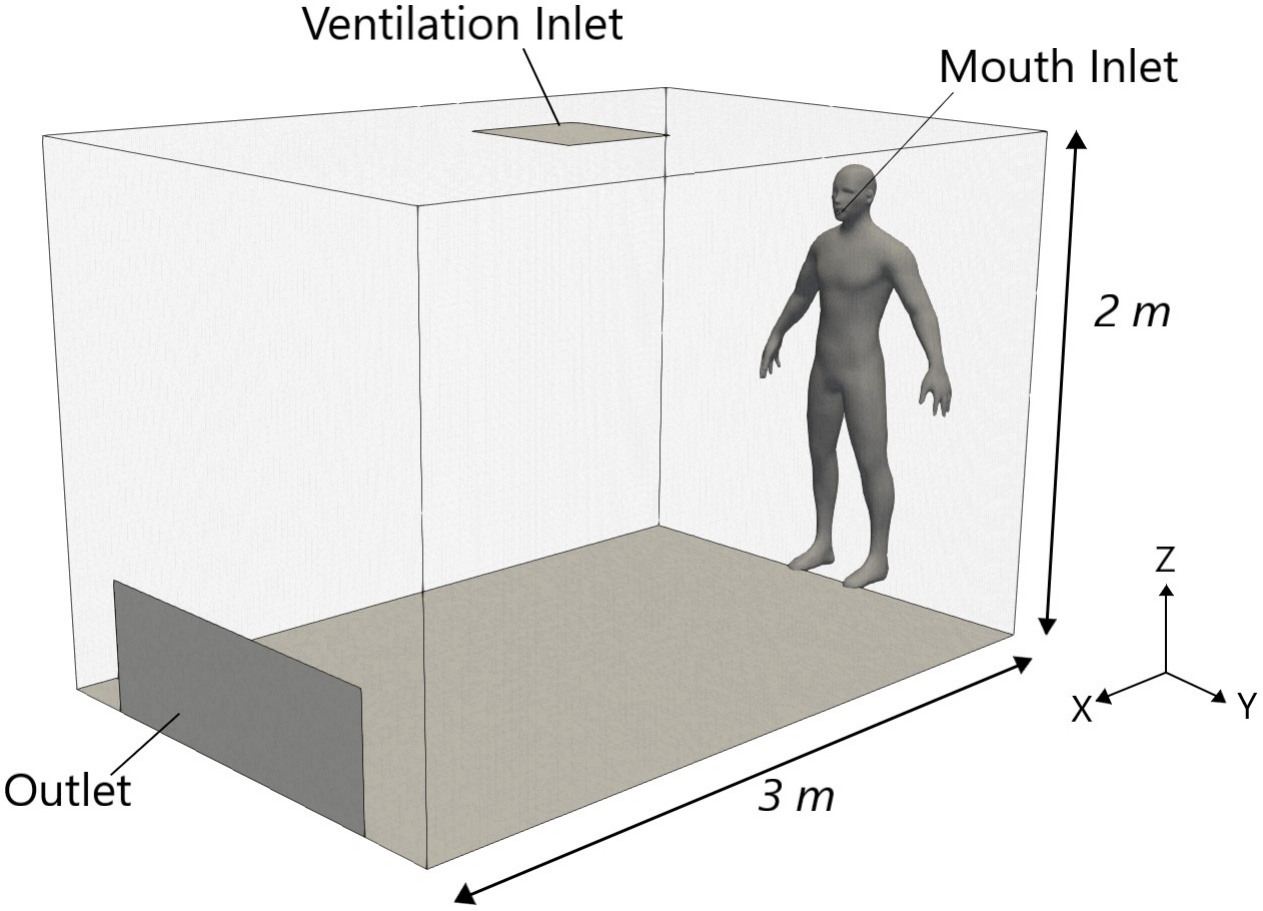
Computational domain. Schematic of room used in CFD simulations. Note the human mannequin is included for visualisation purposes only and is not included in the mesh region.

The computational domain represented a 3 × 2 × 2*m*^3^ room with an air inlet on the ceiling (0.5 × 0.5*m*^2^) and outlet vent on the far wall (0.14 × 0.5*m*^2^) to allow for air recirculation (Fig 1). The mouth was represented by a circular inlet of 0.015 m in diameter that is “stitched” onto the near wall (at X = 0) at a height of 1.57 m, typical of a standing person. In terms of analysing the long-range particle behaviour, the interaction between the exhalation jet and head/body geometry is minor in comparison to the interaction between the exhalation jet and the bulk room air and so the simplified geometry was considered as a reasonable assumption, while also reducing computational cost. The hex-dominant computational grid consisted of 2,261,848 elements. A cut-cell meshing technique was implemented to create increased refinement in the near-mouth region, with a minimum element size of 2.15 × 10^−4^ mm. This allows the flow field in the area of interest to be simulated with a high degree of accuracy.

#### Physical model

A Reynolds Averaged Navier Stokes (RANS) approach was taken to modelling the turbulent flow field of the continuous phase. The k—*ω* SST turbulence model was implemented to solve the compressible RANS equations, based on its robustness in modelling both adverse pressure gradients and flow separation [22]. The continuous phase was modelled as a single-phase ideal gas, based on the properties of air. Specific heat capacity and dynamic viscosity were assumed constant. The respiratory particles were modelled as spherical water droplets, with constant volume and no phase change occurring. The motion of the droplets was determined by Lagrangian Particle Tracking (LPT) to calculate the effects of the gravity force and the drag force caused by the surrounding air. The drag force was obtained from an empirical correlation for a spherical particle and the gravitational force from the density difference of the phases and the droplet volume.

#### Boundary and initial conditions

The inlet velocity at the mouth opening was altered between cases, with a fixed temperature of 34°C and turbulence intensity of 10%. The ceiling, floor and walls of the room were defined as no-slip walls. The outlet vent was specified as a pressure outlet with a fixed value set to atmospheric pressure (101,325 Pa) and back-flow prevented. The internal field had zero initial velocity, atmospheric pressure and a temperature of 23°C.

The distribution of exhaled particles by healthy individuals has been reported to range from 1—145 *μm* [23, 24] for breathing and coughing. Recent data indicates that droplet distributions increase with use of HFNO [25] with droplet diameters from 50—5000 *μm* reported In the present simulations particles were injected with zero initial velocity in a circular region 3 mm in front of the mouth opening. 3000 particles were added over a 0.01 second duration upon initiation of the exhalation jet. A Rosin-Rammler distribution was used to approximate the droplet diameter spread, with a range of 10—200 *μm* and a mean diameter of 70 *μm*. Very small droplets will likely evaporate almost immediately (< 10 *μm*) are neglected.

#### Solution

The *sprayFoam* solver was employed in OpenFOAM to obtain a transient solution for the simulation. This solver uses the PIMPLE algorithm to solve the continuity, momentum and energy equations for the continuous phase, while the Lagrangian Particle Tracking (LPT) method is used to calculate the position and velocity of the discrete particle phase.

A first-order implicit scheme was used discretize time derivatives while second-order schemes were used to discretize spatial derivatives in the continuity, momentum and energy equations for both continuous and discrete phases. The maximum Courant number was set to 0.9 and an automatic time step condition implemented so as to adjust the time step based on the fluid and particle velocity. This yielded a range of time steps based on the inlet velocity magnitude, typically between 5 × 10^−6^ and 5 × 10^−5^ s.

Parallel computing was used for running the final simulations. Decomposition of cases and running of simulations was carried out using 32 processor cores on the Sonic HPC cluster using OpenFOAM v7. The UCD Condominium on ICHEC’s KAY supercomputer was also utilised in the development stage of the model. The total computational time of a case varied between 6.5—87 hrs, with longer duration and higher velocity inlet jets demanding the higher runtime.

### CFD model validation

#### Grid independence study

A grid independence study using the grid convergence index (GCI) method was carried out to quantify the order of accuracy and discretisation error associated with the mesh [26]. Three computational grids of increasing cell density were used, with a grid refinement factor of 1.71. 10 points across vertical lines at 1.5, 6 and 12 cm from the mouth inlet at t = 2s were used for comparison. The global average order of accuracy was found to be 2.08. The mean discretisation error for velocity were to were found to be 5.53% and 2.78%, between the coarse/medium and medium/fine meshes respectively, which motivated the choice of selecting the medium mesh with a maximum element size of 200 microns. due to its relatively low discretisation error and lower computational time vs. the finer mesh.

#### Particle trajectory validation

**Fig 2:**
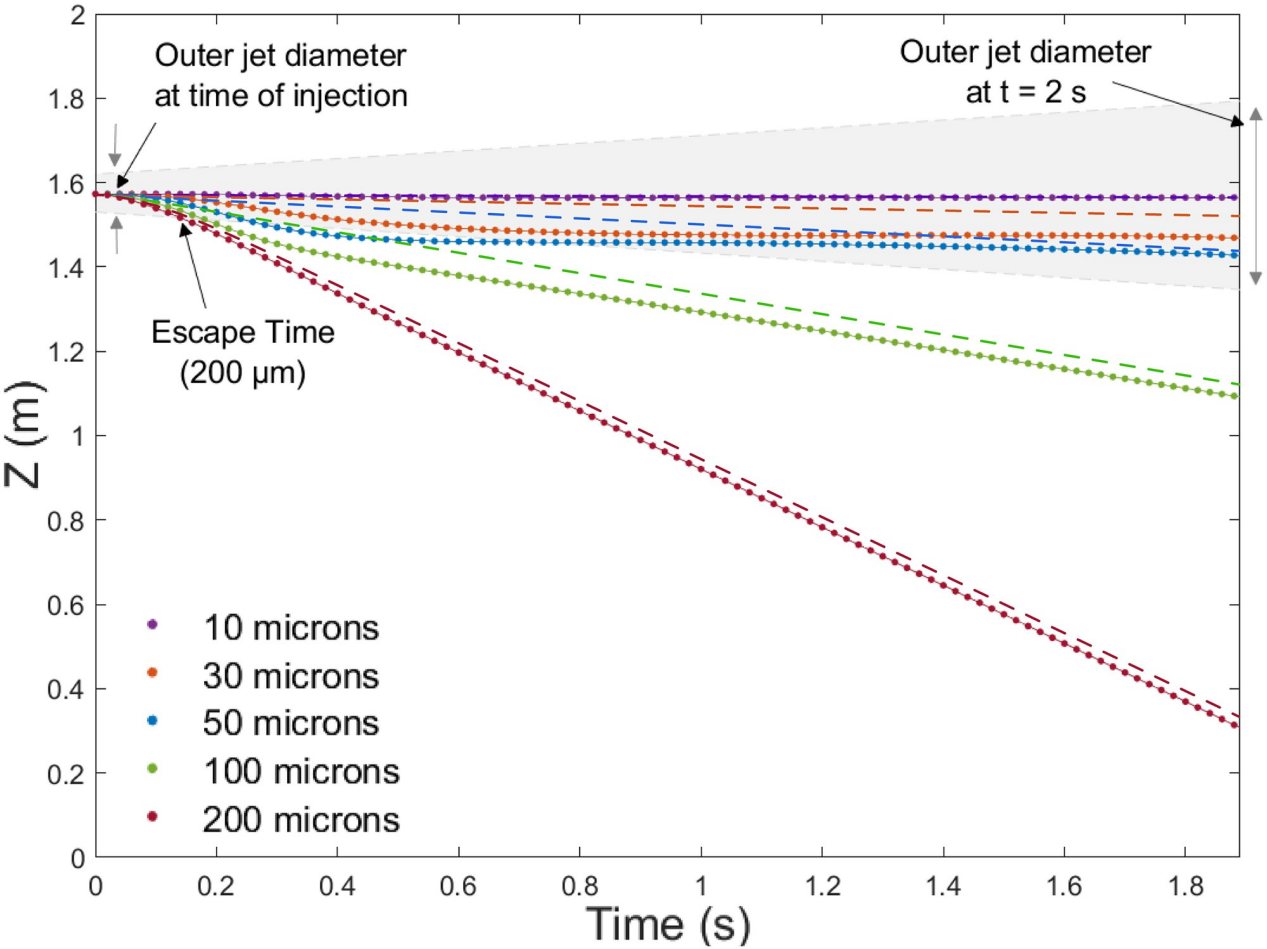
Particle trajectories. Mean vertical travel distance of particles of different sizes over a 2 s interval after injection in a 10 m/s jet. Dashed lines indicate the predicted particle trajectories based on a simple drag model.

Validation of the particle dynamics was carried out by monitoring the trajectories of a range of particle sizes (10, 30, 50, 100 and 200 *μm*) in a constant velocity jet at 10 m/s over a 2 s time frame. The data was compared to a drag model for spherical particles [27] that is valid for a wider range of Reynolds numbers than Stokes drag. Fig 2 shows the mean vertical travel distances of the particles, with the shaded region representing the outer diameter of the exhalation jet. Dashed lines indicate the predictions of the drag model in stagnant air. It is observed that the jet disrupts the vertical transport of the larger droplets until they leave its influence. The 10*μm*—50 *μm* particles remain under the influence of the jet up to 2*m* from their source. Comparison to the results obtained by in a similar study [28] indicated the results were in good agreement, with larger particles (100—200 *μm*) escaping from the jet within 0.2 s and smaller particles (10—50 *μm*) remaining entrained in the jet.

### CFD parametric study

The parametric studies carried out using the validated model were designed based on the two main findings from the schlieren study. The velocity of the exhalation jet increased for both breathing and coughing with the application of HFNO, and the duration of the exhalation period of the breathing cycle was extended. The first study examined the effect of increased jet velocity and exhalation duration during breathing on axial dispersion distance of small to medium sized droplets. The baseline breathing jet velocity of 2 m/s was set based on the peak exhalation flow during a typical breathing cycle [13, 29] and the baseline exhalation duration was set as 2 s [29]. Values of 3 m/s and 5 m/s were selected to represent “slight increase” and “worst case” scenarios for the increase in jet velocity due to HFNO application. To examine the effect of exhalation duration, the jet duration was increased to 3 s, as deemed appropriate from the experimental data analysis.

The second study examined only the effect of a velocity increase on the particle dispersion associated with coughing. The widely accepted value for the cough velocity is of the order of 10 m/s [30, 31]. Stepwise values in the range of 8—14 m/s were chosen to examine a reasonable level of increase in velocity that HFNO application may impart. The duration of the cough was set to 0.5 s, which is at the higher end of the 0.2—0.5 s reported range [32]. Full case details are outlined in Table 1.

**Table 1:** Summary of simulated cases.

Case	Jet Velocity	Duration	Run Time	Droplet Diameter (Range)
Breathing: Standard velocity, standard duration	2 *m*/*s*	2 s	6 s	70 (10—200) *μm*
Breathing: High velocity, standard duration	3 *m*/*s*	2 s	6 s	70 (10—200) *μm*
Breathing: Very high velocity, standard duration	5 *m*/*s*	2 s	6 s	70 (10—200) *μm*
Breathing: Standard velocity, long duration	2 *m*/*s*	3 s	6 s	70 (10—200) *μm*
Breathing: High velocity, long duration	3 *m*/*s*	3 s	6 s	70 (10—200) *μm*
Breathing: Very High velocity, long duration	5 *m*/*s*	3 s	6 s	70 (10—200) *μm*
Coughing: Low velocity	8 *m*/*s*	0.5 s	5 s	70 (10—200) *μm*
Coughing: Standard velocity	10 *m*/*s*	0.5 s	5 s	70 (10—200) *μm*
Coughing: High velocity	12 *m*/*s*	0.5 s	5 s	70 (10—200) *μm*
Coughing: Very high velocity	14 *m*/*s*	0.5 s	5 s	70 (10—200) *μm*

## Results

### Experimental results

**Fig 3:**
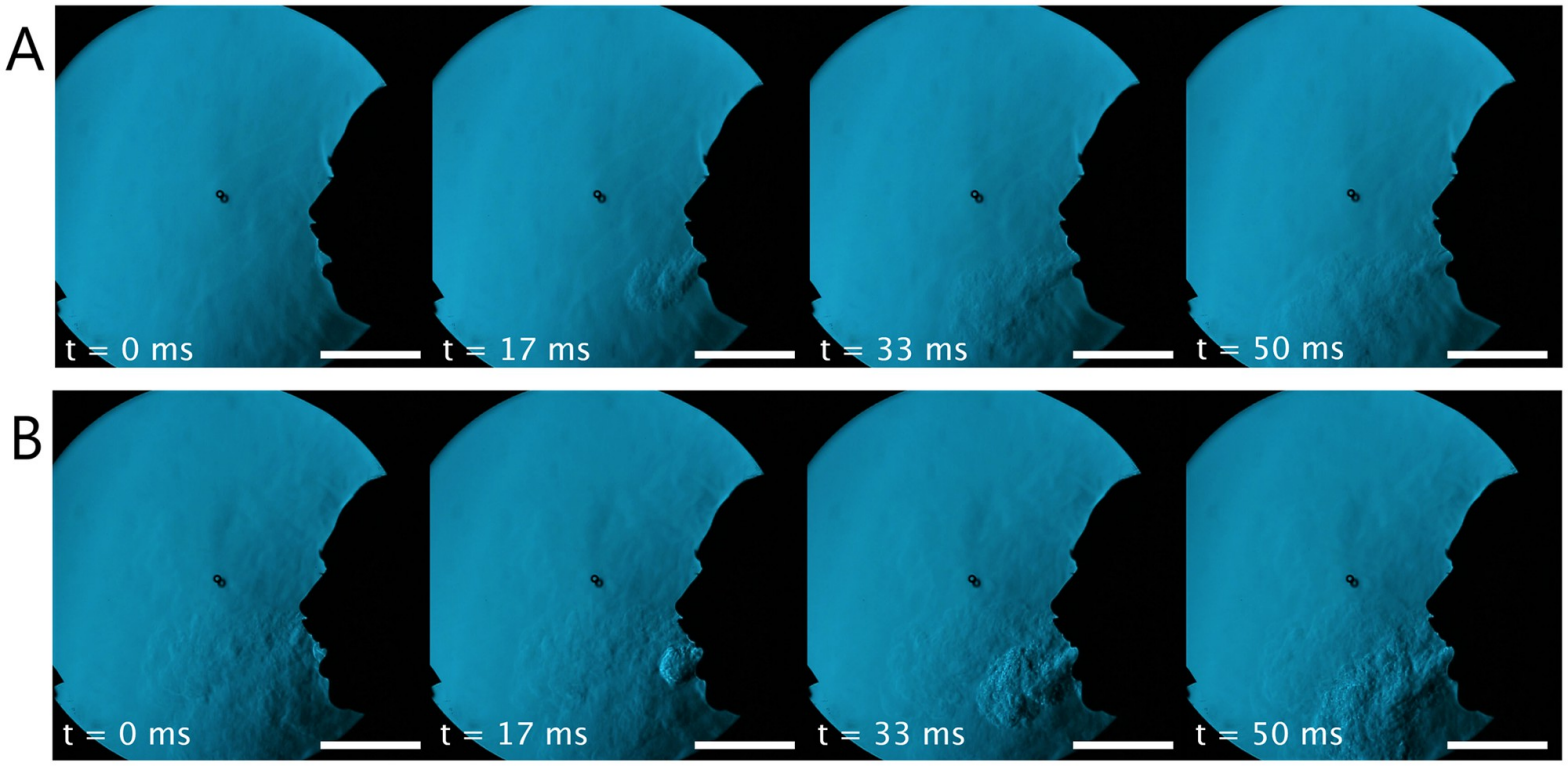
Schlieren coughing comparison. Evolution of a cough for (A) unassisted breathing and (B) HFNO at 60 L/min. The scale bar indicates a length of 0.1 m.

A qualitative analysis of the visual results obtained from the schlieren imaging indicates that the velocity for both breathing and coughing increase notably with HFNO application. Fig 3 shows the development of a cough for (A) unassisted breathing and (B) with HFNO at 60 L/min. There is clear visual evidence of a higher energy associated with cough at 60 L/min HFNO, demonstrated through the increased prominence of turbulent eddies and a notably more “jet-like” appearance to the exhaled flow. These features were observed for all HFNO flow rates and are shown best through the schlieren footage included in the support information (see S1, S2 and S4 Videos). A spherical vortex at the cough jet front can also be observed in Fig 3B, followed by a turbulent quasi-steady state jet in its wake. This finding strongly supports previous proposals to model the human cough as an impulsively-started turbulent jet [31]. This was particularly evident for the cough jets with HFNO at 30—60 L/min, where the vortex ring remained distinct in the flow field for a longer period of time than that of unassisted flow.

**Fig 4:**
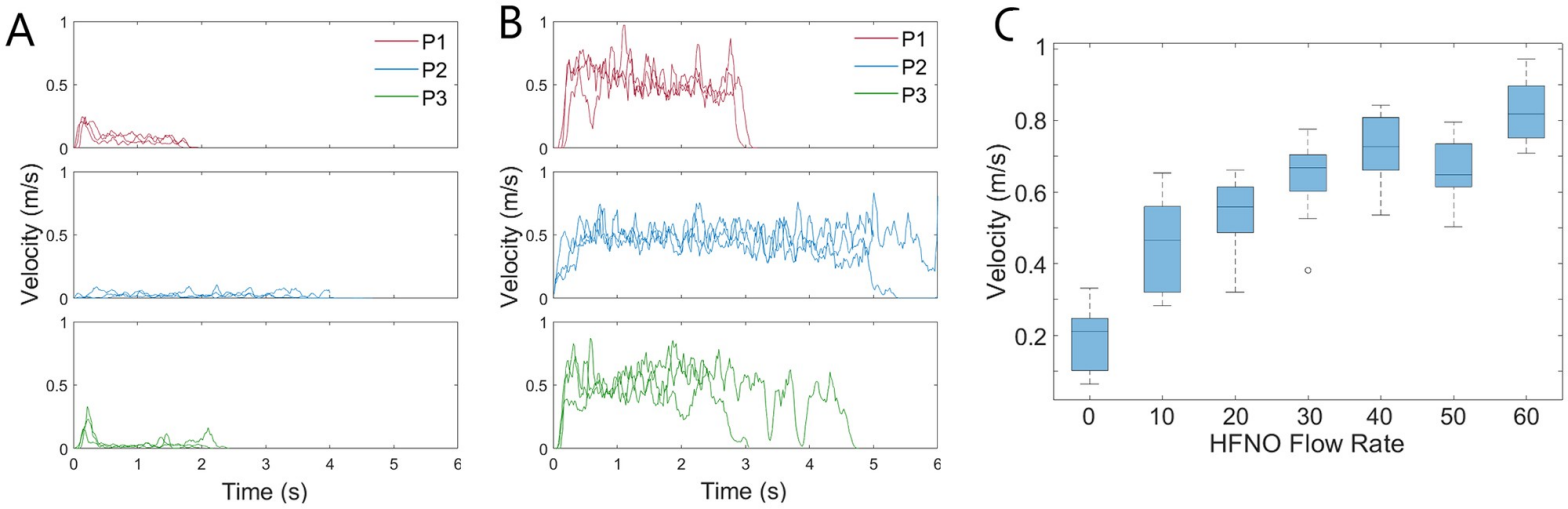
Schlieren breathing trials. Velocity during a single breathing cycle for (A) unassisted breathing and (B) HFNO at 60 L/min for each participant. (C) Range of maximum breathing velocities for all participants. Box and whisker plots show median and interquartile ranges for the data set.

The results of the data analysis using the optical flow Färneback method further indicate the velocity of the exhalation jet increases with application of HFNO therapy for both breathing and coughing. Fig 4 shows the filtered velocity profile for individual breathing cycles for the three participants at the mouth exit, for unassisted breathing (A) and HFNO at 60 L/min (B). Each individual graph shows three separate exhalation cycles overlaid for that participant at the given HFNO flow rate. The three different coloured plots correspond to the three different participants. A significant increase was observed in the exhalation velocity for all applied HNFO flow rates compared to that for unassisted breathing, as documented in the summary results in Table 2. Fig 4C graphically shows these overall ranges and median values, demonstrating that the velocity magnitude of the exhalation jet at the mouth increases with an increasing HFNO flow rate.

**Table 2:** Summary of schlieren imaging data analysis results.

Flow Rate	Breathing Velocity	Cough Velocity
0 L/min	0.21 (0.06—0.33) m/s	0.27 (0.12—0.34) m/s
10 L/min	0.47 (0.28—0.65) m/s	0.62 (0.15—1.08) m/s
20 L/min	0.56 (0.32—0.66) m/s	0.68 (0.13—0.82) m/s
30 L/min	0.67 (0.38—0.78) m/s	0.75 (0.24—1.01) m/s
40 L/min	0.73 (0.54—0.84) m/s	0.81 (0.37—1.05) m/s
50 L/min	0.65 (0.50—0.80) m/s	0.75 (0.42—1.30) m/s
60 L/min	0.82 (0.71—0.97) m/s	0.74 (0.47—1.16) m/s

Median velocity and value ranges at the mouth outlet for breathing and coughing at a range of HFNO flow rates.

A second observation from Fig 4 is that HFNO therapy affects the duration of the exhalation cycle. The duration of the individual breathing cycle differs between the participants, with participants 1 and 3 having a natural exhalation length of about 2 seconds and participant 2 of about 4 seconds. This is extended to 3 seconds on average for participant 1 and 5—6 seconds for participant 2 when HFNO is applied. This yields an approximate 1—2 second increase in exhalation duration.

**Fig 5:**
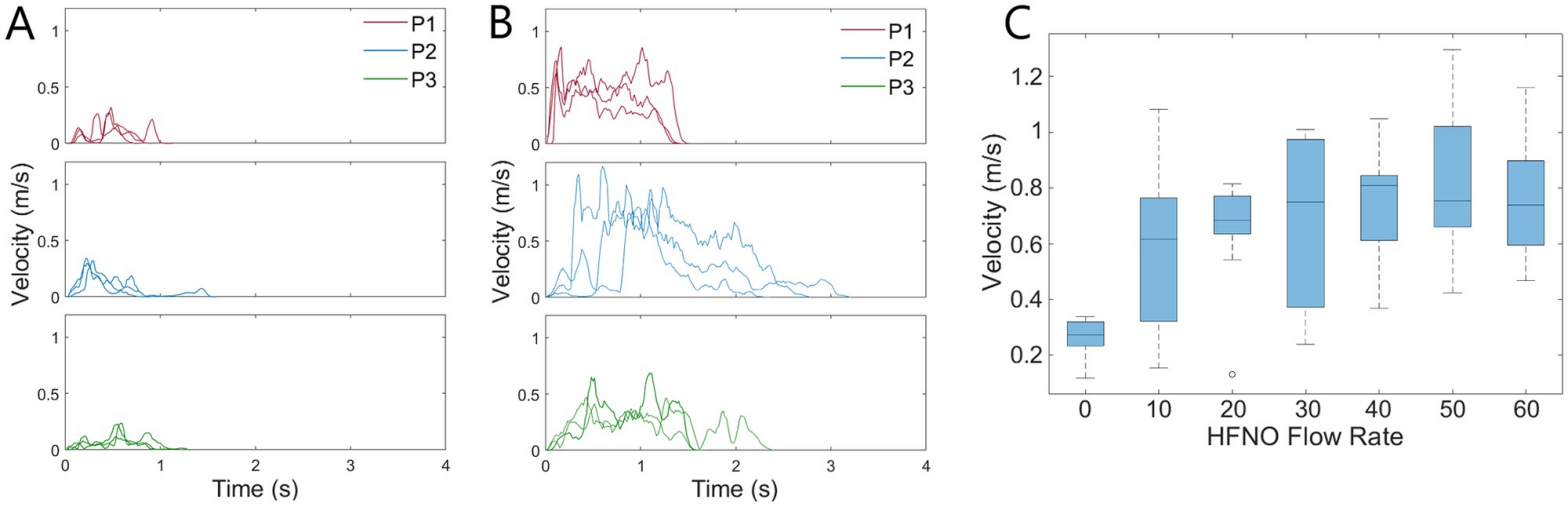
Schlieren coughing trials. Velocity during a single cough for (A) unassisted breathing and (B) HFNO at 60 L/min for each participant. (C) Range of maximum cough velocities for all participants. Box and whisker plots show median and interquartile ranges for the data set.

The findings were similar for the schlieren coughing trials, as shown in Fig 5. The filtered data indicates that standard coughs for all participants have relatively similar profiles, with a mean velocity of 0.27 m/s and a duration of about 1 s, although only a small portion of this is spent at the “peak” velocity (see Fig 5A). The application of HFNO at all flow rates causes an evident increase in the cough velocity, particularly for participants 1 and 2 (see Fig 5B). The velocity profile also shows an increased level of noise, even for the filtered data shown, which is characteristic of the turbulent eddies seen in Fig 3. As seen in Fig 5C, the variation in the velocities measured was large and notably more so than those recorded for breathing. It has been noted in the literature that there is large person to person variability for coughing, caused by differences in initial cough velocity, opening area of the mouth and cough duration [33].

### CFD model results

**Fig 6:**
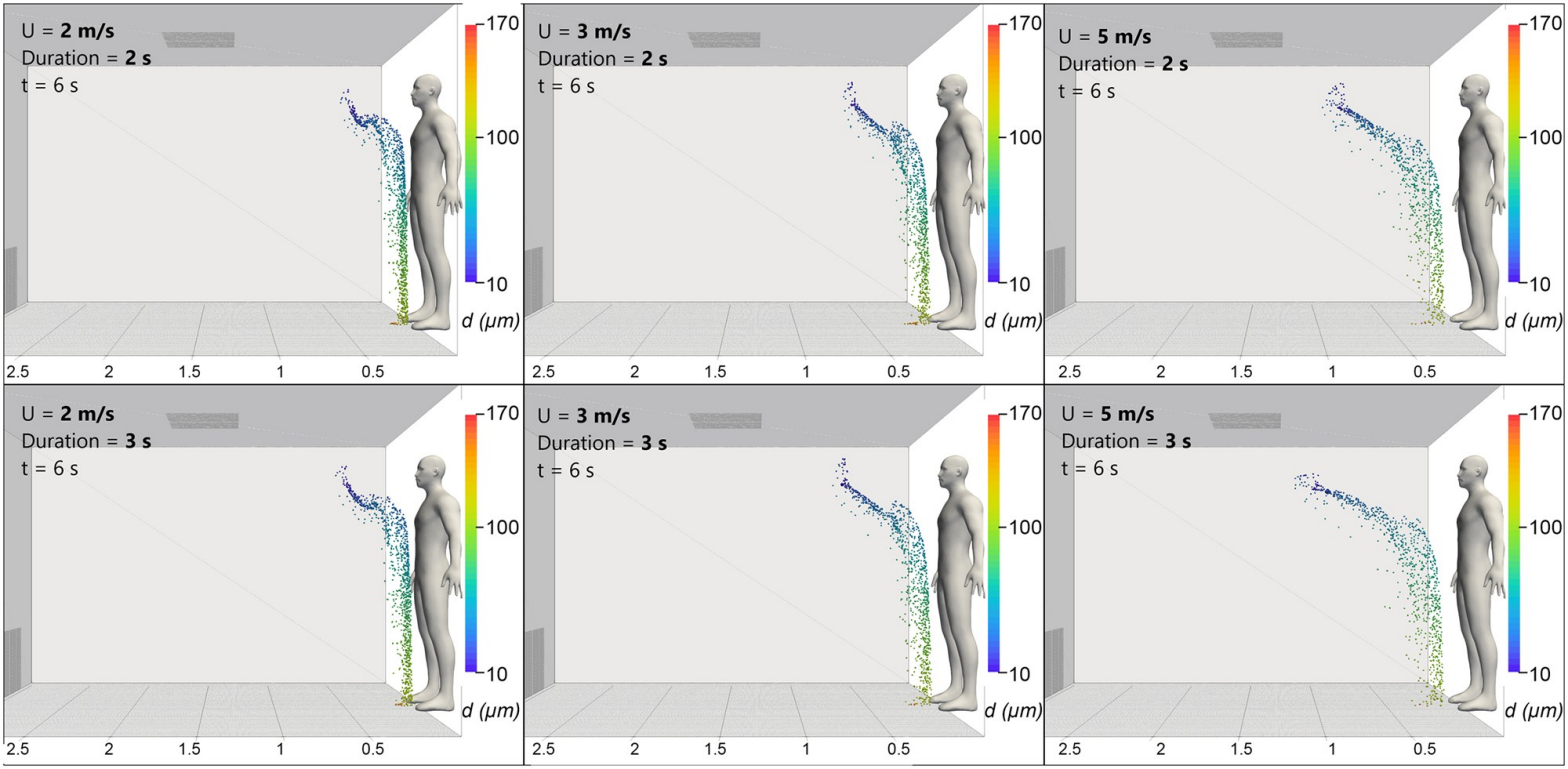
CFD simulations: Breathing. Comparison of droplet cloud shape and positions at t = 6 s after exhalation initiation for a range of exhalation velocities and durations as shown.

Fig 6 shows a visual comparison between the six different breathing cases investigated in the CFD parametric study at 6 s after exhalation begins. The axial distance reached by the droplets increases with both exhalation velocity and duration. The increasing jet velocity causes a “stretching” effect to the droplet cloud, as can be seen by comparing the 2 m/s and 5 m/s exhalations (top far left and far right, respectively). This elongation can also be seen if the duration of the jet is increased and is most obvious for the 5 m/s exhalations (far right top and bottom, respectively). S3 Video included in the supporting information shows the difference in the development of exhalation clouds based on both duration and velocity.

**Fig 7:**
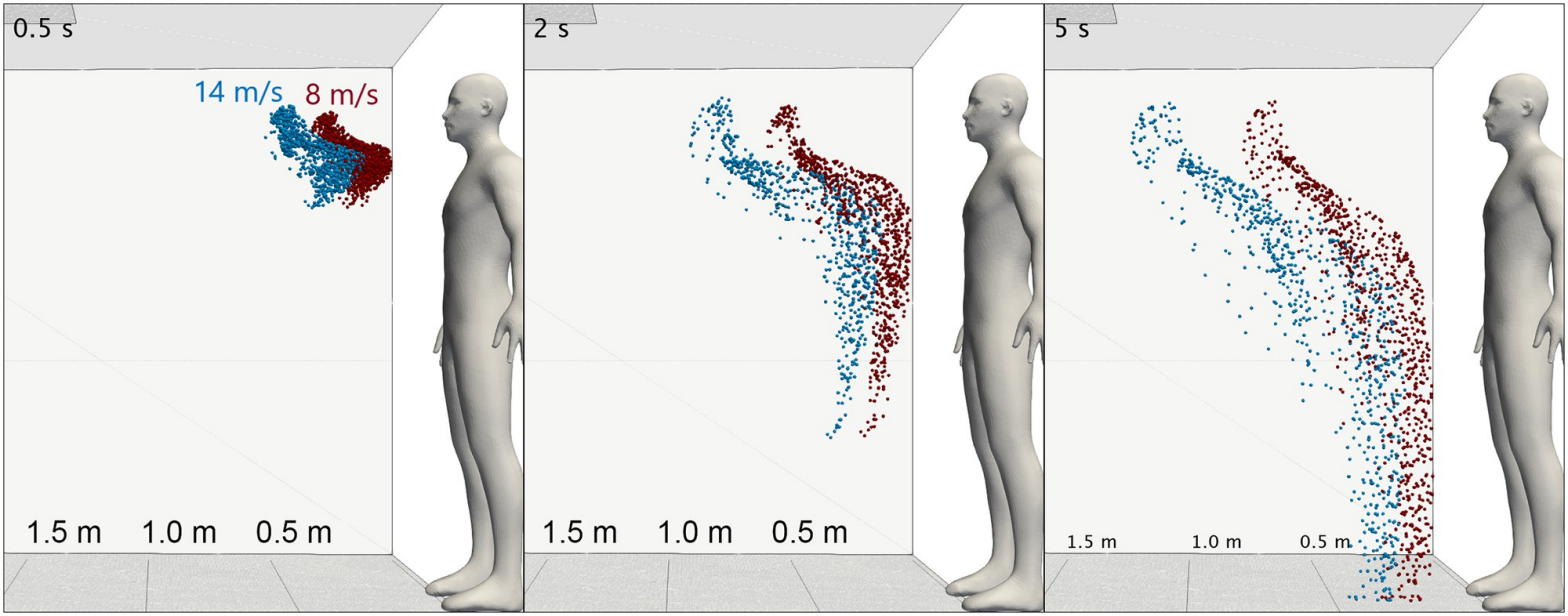
CFD simulations: Coughing. Comparison of droplet cloud shape and positions at t = 0.5, 2 and 5 s for a low velocity (red) and high velocity (blue) cough.

A similar “stretching” effect caused by the increased velocity is observed for the cough cases. Fig 7 shows the development of the weakest (red) and strongest (blue) coughs investigated over a 5 s period.

**Fig 8:**
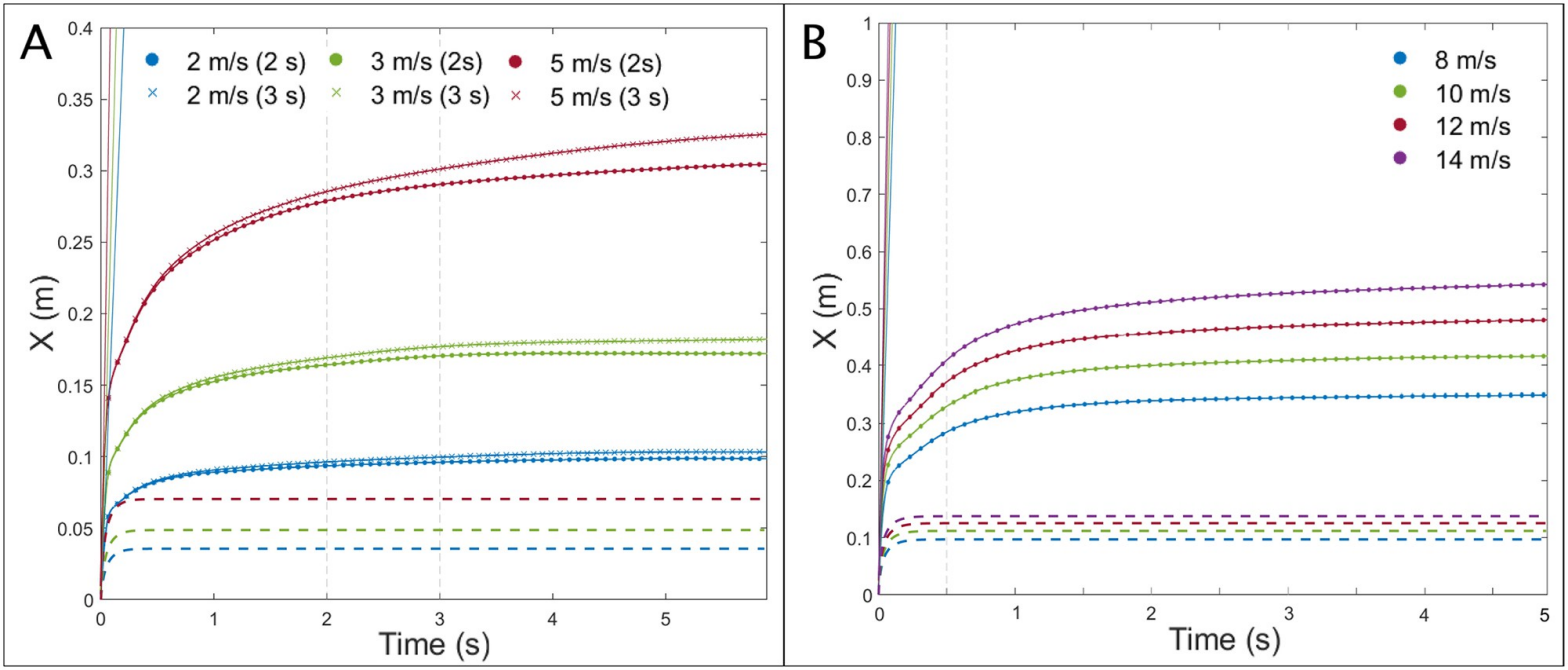
CFD simulations: Particle axial travel distance v time. Mean axial travel distance of droplets for (A) breathing over a 6 s interval after exhalation begins and (B) coughing over a 5 s interval after cough begins. The ballistic (solid lines) and stagnant air resistance (dashed lines) are also shown.

Fig 8A shows the mean distance of travel of all droplets over the 6 s interval simulated for breathing. This is compared to the ballistic and stagnant air resistance conditions. These curves represent scenarios when the droplets are entrained by the jet or have subsequently dropped out. The earlier flattening out of the curve for the 2 m/s jet corresponds to the earlier escaping of the medium to larger droplets from the jet, as seen in Fig 6, compared to those in the 5 m/s jet. The more curved appearance of the 5 m/s jet corresponds to the “stretching” effect noted earlier. The results indicate the axial travel distance is increased by about 6 cm for each 1 m/s increase in breathing velocity for the cases investigated. The growing significance of exhalation duration with increased exhalation velocity can also be noted. A similar profile is observed for the coughing simulations, as shown in Fig 8B. In this case, the stepwise increase in the mean axial travel equates to ∼ 6.5 cm per 2 m/s increase in jet velocity. Note also the significant decline in increasing axial travel after the cough event ends at 0.5 s, as indicated by the dashed vertical line.

**Fig 9:**
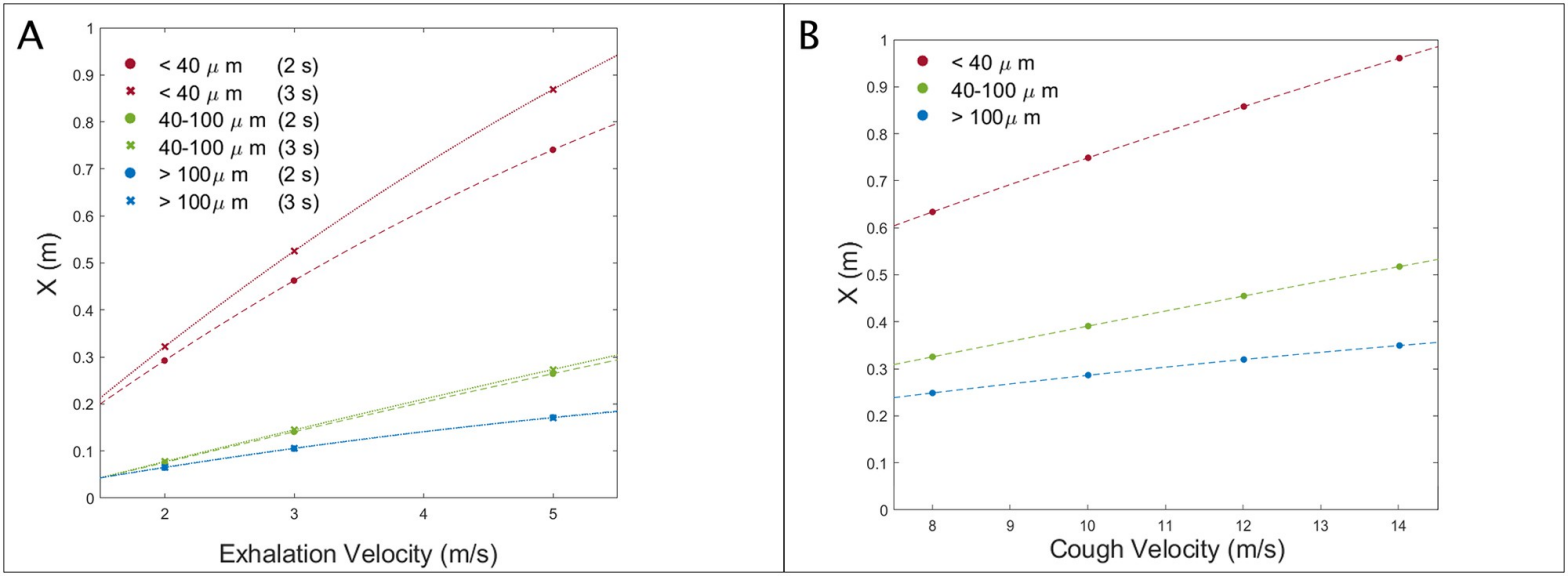
CFD simulations: Particle axial travel distance by diameter. Mean axial travel distance of specified size ranges of droplets for (A) breathing at 6 s after exhalation begins and (B) coughing at 5 s after cough begins.

Fig 9 shows the effect of increasing exhalation velocity (A) and cough velocity (B) on particle travel based on different size ranges. The distances shown correspond to the mean travel distance for the given size range at t = 6 s after exhalation begins (A) and t = 5 s after cough initiation (B). The graph indicates that the dispersion effect becomes more pronounced for smaller droplet sizes as indicated by the steeper slope of the < 40 *μm* line versus the more gradual slope of the > 100 *μm* line. The effect of exhalation duration on axial travel distance in the case of breathing is also considerably more pronounced for the smaller droplets. While the curves for 2 s and 3 s duration essentially overlap for > 100 *μm* droplets (blue), the gap between the 2 s and 3 s duration curves for droplets < 40 *μm* (red) is notable. The full list of figures for the mean and maximum travel distances of the particles by diameter range can be found in the S1 Table.

The results indicate that a 1 m/s increase in the exhalation velocity from 2 m/s to 3 m/s, causes a 58% increase in the mean distance of travel of these smaller droplets (from 29.23 cm to 46.25 cm). A combined increase in the velocity and duration from 2 m/s to 3 m/s and 2 s to 3 s, respectively, causes a 79.6% rise in the mean travel distance (from 29.23 cm to 52.50 cm). A more dramatic increase from 2 m/s to 5 m/s increases the mean travel distance of smaller droplets by 154% (to 74.06 cm). The maximum travel distance of droplets only reaches over 1 m in the worse-case scenario, with a 5 m/s velocity and 3 s duration. However, it is unlikely exhalation velocities will reach 5 m/s in most cases.

In the case of coughing, a 2 m/s increase in cough velocity causes a ∼ 10 cm mean increase in the < 40 *μm* droplet travel. On the other hand, the equivalent mean increase for > 100 *μm* droplets is only ∼ 3—4 cm per 2 m/s velocity increase. The reduced effect of an increased cough velocity vs. an increased breathing velocity may be attributed to the shorter duration of the coughing event. However, comparing the weakest and strongest coughs shows that the 14 m/s cough causes a 47% increase in the maximum distance reached by the smallest droplets within 5 s compared to that for the 8 m/s jet, with distances of 1.185 m, and 0.808 m respectively.

## Discussion

This study addressed the issue of increased aerosol dispersion caused by the application of HNFO therapy using a combination of experimental and modelling methods. This is important as the studies carried out on the therapy, and its potential to increase the risk of airborne transmission of diseases, are limited. The main contributions of this work are therefore twofold. Firstly, a well-established imaging technique was used to visualise the exhalation jet during HFNO application across a full range of flow rates. Secondly, a CFD model was developed to study the influence of the changes caused by HFNO in the fluid dynamics of the exhalation jet on respiratory droplet dispersion.

The experimental investigation found clear evidence that the application of HFNO therapy significantly alters the flow patterns of the exhalation jet for both normal breathing and coughing. Use of the schlieren imaging technique proved highly successful in its ability to provide striking images of the exhalation flow field, which is of course otherwise imperceptible to the naked eye. The high-quality images obtained revealed a dramatic increase in turbulence in the exhalation flow field when compared to unassisted breathing and coughing. The effect was apparent for even the lowest flow rate trialled of 10 L/min but became increasingly intense for higher flow rates of up to 60 L/min. The results also demonstrated that the duration of the exhalation portion of a typical breathing cycle was substantially increased when HFNO was applied, by approximately 1—2 seconds.

Efforts to quantitatively analyse the exhalation velocity using the schlieren data provided mixed levels of success. The application of the optical flow Färneback method indicated the velocity does indeed increase with HFNO application relative to unassisted breathing. However, comparison to the literature indicates the absolute values of velocity recorded are unlikely to be representative of the advection to the turbulence within the base flow. While these results indicate the mean exhalation velocity for unassisted mouth breathing is 0.21 m/s, the widely accepted view in the literature is 1—2 m/s [13, 29]. From this comparison, it can be assumed that the computed values of the exhalation jet velocity with HFNO application is also underestimated. A similar conclusion can be made for the computed cough velocities. For the unassisted cough, the peak cough velocity was estimated to be on average 0.27 m/s, which is an order of magnitude lower than the generally accepted value of 10 m/s [32]. However, the results clearly indicate that the relative level of turbulence and jet velocity at the mouth increases for all applied HFNO flow rates. The mean velocity increase is estimated to be 2.2—3.9 times that for a standard exhalation and 2.3—3 times that for a standard cough.

As for the CFD model, the main contributions of this work lies in the detailed analysis of the transport characteristics of respiratory droplets in turbulent jets with a range of different velocities and durations to mimic the effect of HFNO application on common exhalation activities. Despite a number of physical simplifications, validation by comparison to experimental data indicates the model provides a good indicator of the dispersion patterns of respiratory droplets in the exhalation flow field.

The findings from the CFD model indicate that the increased velocity and, in the case of breathing, increased duration of exhalation yield a greater axial dispersion of droplets. The “stretching” effect on the exhaled cloud of droplets was most pronounced for smaller droplets, in the range of 10—40 *μm*. These smaller droplets remain suspended in the jet and recirculate therein, while larger particles are less affected and continue to follow a ballistic trajectory [34]. The CFD study suggests that the approximate 1 m/s increase in exhalation velocity and 1 s increase in duration when HFNO is applied can cause an 80% increase in mean axial travel distance of these small particles. The findings were similar for cough jets, where increasing the cough strength from “weak” (8 m/s) to “strong” (14 m/s) yields a 47% increase in the maximum distance of small particle travel. Moreover, these smaller droplets remain suspended in the zero-velocity ambient air 4—5 s after the exhalation jets cease. These smaller droplets have very low settling velocities (< 0.1 m/s, [28]) and are highly susceptible to ambient velocity flow patterns, meaning they are likely to remain suspended in the air for long periods of time. Since particles of 10 *μm* and below can be inhaled and have various health implications [35], the increased risk posed to healthcare workers is non-negligible.

The main limitation of the experimental work was in the limited field of view attainable using the schlieren set-up. The mirror diameter used in the study was 0.4 m, meaning the far-field region of the flow could not be imaged. The momentum effect dominated in the region of the jet examined, but it is expected buoyancy forces would prevail in the outer region. This can have a significant effect on the vertical transport of small aerosols that remain entrained in the exhaled air at long distances from the source [34]. However even with a wider field of view, due to the cooling of the exhalation jet as it moves away from the source, the true distance of the jet is still likely to be underestimated. The limited sample size should also be acknowledged. There is huge variation in exhalation patterns and relative cough strengths between individuals and therefore additional participants should be included in future studies to identify trends in the flow patterns of HFNO over a broader range.

Experimental and numerical uncertainties must also be addressed. Uncertainty around the velocity estimation method were mitigated by deferring to reported velocity ranges from the literature. It is expected that better velocity estimation is possible at higher frame rates so that the turbulent flow structures can be tracked over smaller distances. There also exist sources of uncertainty when modelling two phase flows [36]. Herein we assume that there is no significant evaporation in the length and time scales considered, however variation in evaporation rate can play an important role.

More violent respiratory events such as coughing and sneezing are well-studied from an engineering point of view [14–16, 23, 31, 32, 34]. The argument has been made that greater attention needs to be directed towards the more frequent continuous processes of breathing and speaking as these are not treated with the same discretion as the evident clinical indicators of illness like coughing and sneezing [37]. This is particularly true in the case of HFNO due to its combined effect to increase flow rate and duration of exhalation. A recent study measuring aerosol generation during HFNO application showed there was no notable increase in particle concentration with the therapy [38]. However, healthy volunteers took part in the study and it has been proven that respiratory health influences the nature of respiratory particles produced, meaning sick individuals generally produce a greater volume of aerosol particles [39]. Although HFNO therapy may not be the root cause of increased particle generation, the larger volume produced by ill patients will inevitably be transported further due to the effects of the therapy on the exhalation characteristics. The study also indicated that mean droplet diameter decreased with increased HFNO flow rate [38]. As stated previously, these smaller droplets have a greater ability to remain airborne and penetrate the airways [35]. These combined effects undoubtedly put healthcare workers at increased risk when in close proximity to patients.

The results of this study further exemplify the necessity of taking sufficient “airborne precautions” as advised by the WHO, including wearing appropriate PPE and ensuring adequate ventilation [1]. A recurring recommendation in the literature to limit the aerosol dispersion is the wearing of a simple surgical mask by the patient over the HFNO nasal cannula [7, 10, 12]. Although the use of face masks to prevent the dispersion of respiratory jets has been proven in recent studies using both experimental methods [40] and CFD simulations [15, 17], the safety of mask application to sick patients receiving HFNO therapy remains a cause of concern [41]. Alternative mitigation strategies are therefore of great interest to ensure this therapy can be utilised while ensuring the safety of hospital staff.

## Conclusion

As the COVID-19 pandemic continues to pose a serious threat to healthcare systems on a global scale, it is essential that all options to effectively treat critically ill patients are investigated. The role of HFNO treatment in preventing early intubation, improving patient comfort and providing an alternative to mechanical ventilation in resource-constrained settings has been emphasised in the literature. However the potential for HFNO to increase aerosol generation and hence put health care workers at increased risk of viral transmission remains a cause for concern. This study has been successful in outlining an experimental and modelling framework for investigating the infection risk associated with HFNO therapy. Qualitative analysis of schlieren images revealed a highly turbulent exhalation and cough jet when HFNO was applied, while quantitative measures indicated an increased flow rate and exhalation duration with the therapy. The CFD model showed that these highly energetic exhalation jets increased the dispersion distances of expired aerosol, particularly in the case of smaller particles that can remain airborne for long periods. As our understanding of the external flow during HFNO application is still not fully understood, additional experimental, clinical and modelling trials are necessary to estimate the risk of viral transmission associated with the therapy and better control the behaviour of the treatment.

## Supporting information

S1 Video Schlieren imaging: Breathing. A series of video clips showing schlieren images obtained of breathing with HFNO at different flow rates for one participant. Videos are shown at half-speed playback. Raw video footage has been enhanced to emphasize different flow regimes.
(MOV)

S2 Video Schlieren imaging: Coughing. A series of video clips showing schlieren images obtained of coughing with HFNO at different flow rates for one participant. Videos are shown at half-speed playback. Raw video footage has been enhanced to emphasize different flow regimes.
(MOV)

S3 Video CFD simulation: Breathing. A comparison of aerosol dispersion with and without HFNO application, based on an increased flow rate and exhalation duration when HFNO is applied, as observed in the experimental study. Aerosol size and quantity is for visualisation purposes only.
(MOV)

S4 Video HFNO flow visualisation. Simultaneous Schlieren and laser light sheet visualisation of a test subject undergoing high flow nasal oxygen therapy at increasing flow rates. The laser sheet illuminated the subject’s sagittal plane. Aerosol (Aerogen Solo, 5 *μm* saline is delivered via the nasal cannula. The image colour has been partially inverted to improve contrast against a black backdrop. The small particles are observed to travel up to 1.5 m from the subject and larger particles resulting from coughing are observed to fall to the ground).
(MP4)

S1 Table CFD simulations particle travel by diameter. Mean and maximum axial travel distances at 6 s and 5 s for specified droplet diameter ranges in a range of breathing and coughing jet velocities, respectively.
(XLSX)
